# Temperature Effects and Entropy Generation of Pressure Retarded Osmosis Process

**DOI:** 10.3390/e21121158

**Published:** 2019-11-27

**Authors:** Bassel Abdelkader, Mostafa H. Sharqawy

**Affiliations:** School of Engineering, University of Guelph, Guelph, ON N1G 2W1, Canada; babdelka@uoguelph.ca

**Keywords:** pressure retarded osmosis, temperature effect, power density, entropy generation, optimum flow

## Abstract

Pressure retarded osmosis (PRO) is considered as one of the promising and new techniques to generate power. In this work, a numerical model was used to study the effect of the flow streams temperature on the performance of the PRO process and entropy generation. The variation of the feed solution and draw solution temperatures, pressure difference, concentration difference, and flow rates on the power density and entropy generation were discussed. The model results were validated with experimental measurements obtained from literature and showed a good agreement with the model predictions. It was found that the power density increases by about 130% when both feed solution and draw solution temperatures increase from 20 °C to 50 °C. The feed solution temperature has more impact on the power density than that of the draw solution. This is due to the direct effect of the feed solution temperature on the water permeability and diffusion coefficient. The effect of the feed solution temperature becomes significant at higher concentration differences. Whereas, at low concentrations, the power density slightly increases with the feed temperature. Furthermore, it is found that there is an optimum volumetric flow in the channels that maximizes the power density and minimizes the entropy generation when fixing other operating conditions.

## 1. Introduction

Pressure retarded osmosis (PRO) is a method used to generate power when two solutions with different salinities are combined in a controlled process. The salinity gradient will drive the water from the lower concentration (feed) to the higher concentration (draw) through a semi-permeable membrane to reach an equilibrium [1,2,3]. Energy is generated when the draw solution is depressurized using a hydro turbine [4]. The flow of water across the semi-permeable membrane increases as the osmotic pressure difference between the two solutions increases.

PRO can generate a constant power supply for base loads as compared to other renewable energy technologies [5,6,7,8,9,10]. It has the potential to generate 157 GW of clean energy or 1370 TWh/year, taking into consideration the conservative estimations on losses and inefficiencies using only 3730 km^3^/year of the river water discharge, which represents 10% of the global river water discharge. This is equivalent to the electrical consumption of 520 million people, based on the average global electricity [11]. The potential of the PRO system was investigated in many countries. For example, the potential PRO energy was estimated to be in the range of 0.9–10.5 TWh/year in the remote regions of Quebec, Canada [12]. There is no commercial membrane that is engineered or optimized for the PRO process. However, reverse osmotic (RO) membranes have commonly used in previous PRO studies [13,14]. These membranes consist of a thin non-porous layer (active layer) of about 0.2 m thickness, and a thick porous permeable layer (support layer) of about 150 m thickness.

Generally, any dense, non-porous, selectively permeable material can be used as a membrane for the PRO process. However, the membrane characteristics and operating parameters are crucial to the performance of the process. In addition, the membrane properties and diffusion coefficients of water and ions are affected by temperature [15]. The length of the polymer chain in the active layer increases with the temperature, which increases the membrane pore size. Thus, the permeation of water and ions increases with the temperature. Although the permeate water flux increases with the temperature, the rejection of Na and Cl ions slightly decreases. This is due to Na and Cl ions having a smaller hydrated diameter compared to divalent ions, thus, Na and Cl have higher permeation. However, the rejection of divalent ions increases, as the increase in the permeate flux is more than the increase in divalent ions permeation [15,16,17,18].

Fouling is a complex and undesirable phenomenon in osmotic membranes, particularly in the PRO process. This is because the feed stream faces the porous substrate (support layer) in PRO therefore, the foulants are carried into the pores by the permeate flux which make the internal concentration polarization effect worse. The inverse salt flux can also promote fouling as it exaggerate the biofouling by enhancing bacterial extracellular polymeric substances [9]. Fouling is caused by the adhesion of inorganic and organic materials to membrane surfaces and pores, which leads to pore blockage with time. Inorganic fouling is caused by scale formation on the membrane surface, while organic fouling is caused by a combination of microbial and natural organic matter [19,20,21,22]. Higher temperature increases scale formation on the membrane surface due to the effect of temperature on solubility [23]. The scale formation results in homogeneous crystallization and supersaturation at higher temperatures. For instance, calcium carbonate starts to precipitate on the membrane surface at 20 °C [24,25,26] and form hard calcite scales at 25 °C, while aragonite scales start to form at 45 °C [27]. 

The effect of water temperature on the performance of PRO system was investigated by references [28,29,30,31]. Generally, increasing the water temperature enhances the performance of the PRO process. The feed solution temperature has more impact on the performance. This is due to the feed solution temperature having a higher effect on the mass transfer coefficients and water permeability compared to that of the draw solution temperature [30]. However, it increases the ions diffusion which results in a high internal concentration polarization. This changes the structure parameter which is supposed to have a constant value, but it varies with the concentration profile at the active layer–support layer interface [28].

Concentration polarization occurs due to the presence of a salt gradient across the membrane [32]. There are two types of concentration polarization; one that occurs in the vicinity of the membrane surface and is known as the external concentration polarization, while the other occurs inside the membrane and is known as the internal concentration polarization [5,6]. Generally, the concentration polarization decreases the effective osmotic pressure difference across the active layer, however the external concentration polarization could be reduced by increasing turbulence near the membrane surface at the draw solution side. Decreasing the effective osmotic pressure difference lowers the water flux through the membrane, which consistently decreases the power density. The impact of concentration polarization increases with the draw solution salinity [7]. Therefore, considering membrane polarization in any PRO system design is very essential. PRO has an enhanced performance when the support layer faces the feed solution, as it will decrease the internal concentration polarization [5,10].

Entropy generation calculation is an effective method to analyze irreversibilities in processes and to investigate the optimum operating conditions that maximize the produced energy [33]. Irreversibilities in PRO process arise from the pressure drop along the flow channels, and the diffusion of salt ions due to the concentration differences across the membrane. If there is also a temperature difference between the feed and draw solution across the membrane, there will be irreversibilities due to the heat transfer. The increase of the temperature affects the entropy generation as it increases the osmotic pressure of the solutions. The theoretical limit of the maximum generated power from an ideal PRO process depends on the mixing ratio (the mass flow rate of the feed solution to that of the draw solution) and the salinity difference between the feed and draw streams [34]. For a draw solution of 35 g/kg salinity and feed solution as pure water, the maximum specific energy generated is 1 kWh/m^3^ of the feed solution [34]. 

From the previous discussion, it is clear that the solution temperature has an impact on the performance of the PRO system as it affects the membrane properties and the osmotic pressure. However, to the best of the authors’ knowledge, the combined effect of temperature on water and membrane properties as well as the entropy generation was not studied. Therefore, the objective of the present research is to study the combined effect of feed and draw temperatures on the performance of the PRO process using a numerical model that considers all non-idealities. In addition, the effect of pressure difference, streams concentration, and flow velocity on power density and entropy generation are discussed. 

## 2. Mathematical Model

**Figure 1:**
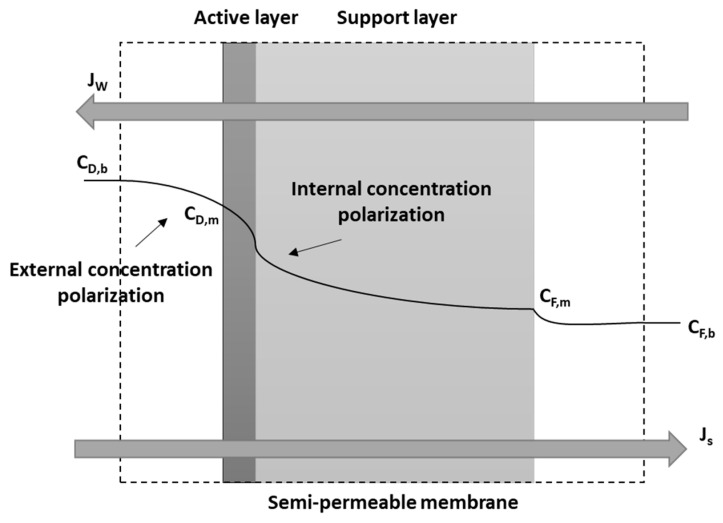
Pressure retarded osmosis (PRO) concentration profile for an asymmetric membrane with the dense layer facing the draw solution.

The solution diffusion model is used to study the effect of feed and draw temperatures on the performance of the PRO process. The osmotic pressure difference is the driving force which generates a transmembrane flow from the feed solution to the draw solution. A reverse salt diffusion occurs from the draw solution to the feed solution due to the concentration gradient across the membrane. The PRO membrane is divided into a number of computational cells along its length, and the diffusion model equations are solved for each cell. The performance of the PRO process is evaluated by calculating the power density, which is the power generated per unit membrane area or the product of transmembrane water flux and pressure difference across the membrane. The osmotic pressure difference is calculated across the active membrane layer to include the effect of the concentration polarization, as shown in Figure 1.

**Figure 2:**
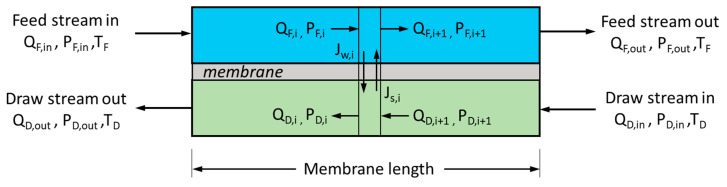
Schematic of a membrane element and a counterflow PRO process.

Conservation of water and salt mass are applied to determine the variation of water and salt concentrations along the membrane. The numerical model divides the membrane into small cells of equal size as shown in Figure 2. The model takes into consideration the effect of the pressure drop and concentration polarization. Moreover, the effect of temperature on the membrane properties and flow streams is considered while neglecting the heat transfer through the membrane. The numerical model is solved assuming a counterflow arrangement of the feed and draw streams. The change of the volumetric flow in the feed and draw channels are given by;

(1) $$dQ_{F}=-J_{w}\;dA_{m}$$

(2) $$dQ_{D}=J_{w}\;dA_{m}.$$

The local water and salt fluxes are determined from the solution diffusion model. The water flux through a membrane is related to the water permeability and the effective pressure difference as shown in Equation (3).

(3) $$J_{w}=A\left(\Delta \pi_{eff}-\Delta P\right)$$

Salts flux is in the opposite direction of the water flux, from the draw solution channel to the feed solution. The salt flux is related to salt permeability coefficient of the membrane active layer and the solute concentrations at the interface of the active and support layers as shown in Equation (4).

(4) $$J_{S}=B\left(C_{D,m}-C_{F,m}\right)$$

The effective osmotic pressure difference can be evaluated from Equation (5). The osmotic pressure is calculated using the correlation given by [35] which is a function of the temperature and salinity. The power density is calculated using Equation (7).

(5) $$\Delta \pi_{eff}=\pi_{D,m}-\pi_{icp}$$

(6) π=f(T,C)

(7) $$W=J_{w}\Delta P$$

The water permeability, *A* depends on feed solution viscosity, pore size, membrane thickness, and porosity [36] as given by Equation (8). The salt permeability is determined by Equation (9) [30].

(8) $$A=\frac{r_{p}^{2}\varepsilon }{8\mu_{F}t_{m}}$$

(9) $$B=\frac{A\beta RT\frac{J_{S}}{J_{W}}}{1+\frac{A\Delta P}{J_{W}}}$$

In PRO, water flows from the feed solution across the membrane to the draw solution, while salt flux from the draw solution to the feed solution. Thus, there is a salt gradient in the vicinity of the membrane surface (external concentration polarization) and inside the membrane (internal concentration polarization). Therefore, the effective osmotic pressure difference for water flow across the membrane is lowered. The transport of salt can be described by [30]

(10) $$C_{icp}=\left(C_{F,m}+\frac{J_{S}}{J_{W}}\right)\exp\left(J_{W}K\right)-\frac{J_{S}}{J_{W}}$$

where *K* is the solute resistivity for diffusion within the support layer and given by Equation (11) [30].

(11) $$K=\frac{\tau t_{m}}{\varepsilon D_{F}}=\frac{S}{D_{F}}$$

The ion concentration is calculated across the active membrane layer to include the effect of the concentration polarization. The ion concentration of the draw and feed solutions at the membrane surface is given by Equations (12) and (13) [30].

(12) $$C_{D,m}=\left(C_{D,b}+\frac{J_{S}}{J_{W}}\right)\exp\left(-\frac{J_{W}}{k_{D}}\right)-\frac{J_{S}}{J_{W}}$$

(13) $$C_{F,m}=\left(C_{F,b}+\frac{J_{S}}{J_{W}}\right)\exp\left(\frac{J_{W}}{k_{F}}\right)-\frac{J_{S}}{J_{W}}$$

where *k* is the mass transfer coefficient in the solution and given by Equation (14), whereas, the Sherwood number (Sh) is calculated using Equation (15) [32].

(14) $$k=\frac{\mathrm{Sh}\;D}{d_{h}}$$

(15) Sh=0.2Re0.57Sc0.4

**Figure 3:**
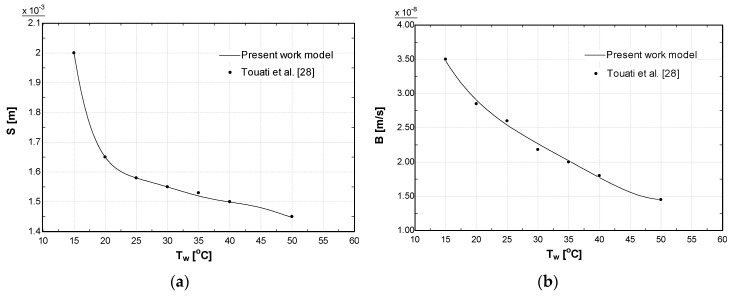
Comparison between the experimental data and the used correlation (**a**) structure parameter, (**b**) membrane salt permeability [28].

The water temperature affects the osmotic pressure and the membrane properties. To include the effect of temperature on the structure parameter and membrane permeability, a correlation based on experimental data for IGB membrane (a cellulose acetate membrane developed by Fraunhofer Institute for Interfacial Engineering and Biotechnology) was used [28]. The correlations were obtained by using a regression analysis of the 7 experimental data points measured in the temperature range of 15–50 °C by [28] and an average temperature for feed and draw was assumed. The resulting empirical correlation for the structure parameter and membrane salt permeability are given by Equations (16) and (17) respectively. The correlations were consistent with the experimental data [28] as shown in Figure 3 with an average deviation of 0.3% and 2.1%, respectively. The IGB membrane properties are presented in Table 1.

(16) $$\begin{array}{ll} S= & 1.47006\times 10^{-2}-0.22595\times 10^{-2}T_{w}+1.60154\times 10^{-4}{T_{w}}^{2}-5.95111\times 10^{-6}{T_{w}}^{3}+\\ & 1.21775\times 10^{-7}{T_{w}}^{4}-1.29945\times 10^{-9}{T_{w}}^{5}+5.64570\times 10^{-12}{T_{w}}^{6} \end{array}$$

(17) $$\begin{array}{ll} B= & 8.30289\times 10^{-8}-5.76329\times 10^{-9}T_{w}+2.28665\times 10^{-10}T_{w}^{2}-4.41125\times 10^{-12}T_{w}^{3}\\ & +3.19064\times 10^{-14}T_{w}^{4} \end{array}$$

The pressure drop in the flow channels due to friction along the membrane length is calculated as follows [37]:

(18) $$\Delta P_{drop}=-\frac{f\rho V^{2}}{2d_{h}}dL$$

where *f* is the friction coefficient given by [37];

(19) f=6.23Re−0.3.

The system is analyzed from the second law of thermodynamic perspective. The water and salt transport across the membrane causes variations in concentration, pressure, and temperature between the feed and draw streams which adds to the system irreversibility and reduces the produced work. The entropy generated in the system represents the amount of irreversibility caused by the transport process. Applying an entropy balance on the PRO process, the rate of entropy generated can be calculated from the inlet and outlet stream specific entropy and flow rates as follows [38];

(20) $${\dot{S}}_{gen}={\left({\dot{m}}_{D}s_{D}+{\dot{m}}_{F}s_{F}\right)}_{out}-{\left({\dot{m}}_{D}s_{D}+{\dot{m}}_{F}s_{F}\right)}_{in}$$

where *s* is the specific entropy, ${\dot{m}}_{D}$ and ${\dot{m}}_{F}$ are the mass flow rate of the draw and feed solutions, respectively. The pressure, temperature, and salinity of each stream contribute to the specific entropy at the inlet and outlet states. The specific entropy of seawater as a function of pressure, temperature, and salinity is calculated using the correlations given by Nayar et al. [35], while the mass flow rates are calculated from the mass balance given by Equations (1) and (2). In addition, the pressure drop in the feed and draw channels determined from Equation (18) is considered for the entropy generation calculation.

## 3. Results and Discussion

The performance of PRO system using IGB membrane is investigated by studying the effect of temperature and operating parameters on the power density and entropy generation. In this regard, the numerical model is first validated with experimental data available in the literature, followed by a parametric analysis. The effect of the applied pressure, solution concentration, and feed and draw solutions temperatures are investigated. In addition, the effect of flow rate was analyzed to determine the optimum velocity. The experimental operating conditions are presented in Table 2. The salinity of the draw solution was 70 g/kg and 35 g/kg, which represents the brine and seawater concentrations. The seawater properties correlations obtained by Nayar et al. [35] were used for calculating the thermophysical properties of the draw solution.

## 4. Model Validation

**Figure 4:**
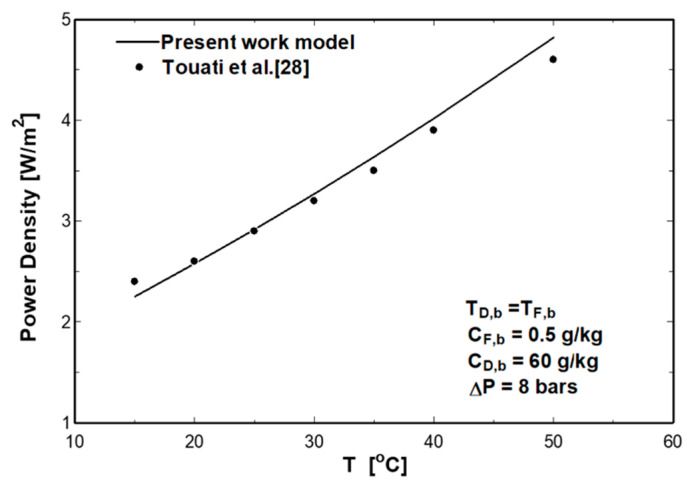
Comparison between the numerical model and experimental data [28].

The numerical model is validated using the experimental data presented by Touati et al. [28]. These experiments were conducted using an IGB membrane with effective membrane area of 18 cm^2^. The IGB membrane characteristics are given in Table 1. The pressure difference was fixed to 8 bars and the fluxes of the feed and draw solutions were 50 mL/min, whereas the salinity of the draw solution was 60 g/kg and the feed solution was 0.5 g/kg. The temperatures of feed and draw solutions were maintained equal but varied in each experimental run within the range 15–50 °C. The present numerical model was used to simulate the performance of the same IGB membrane, and the results are compared with the measured experimental data. Figure 4 represents the effect of feed and draw solutions temperature on the power density. As shown in this figure, the numerical model has a good agreement with the experimental data with a maximum error of 6.5% and an average error of 3%.

### 4.1. Effect of Feed and Draw Solutions Temperatures on PRO Performance

**Figure 5:**
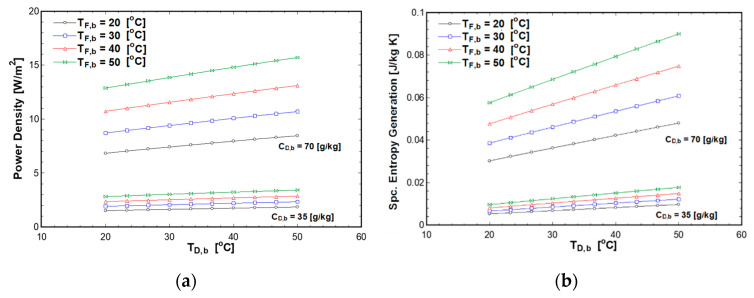
Effect of feed and draw solution temperature on (**a**) power density; (**b**) entropy generation at C_F,b_ 0.5 g/kg.

The effect of the feed and draw solutions temperatures on power density and specific entropy generation is shown in Figure 5. The draw temperature varied from 20 to 50 °C. Meanwhile, the pressure was equal to half of the osmotic pressure difference to simulate the theoretical maximum power density condition. The concentration of the draw solution was studied at 35 g/kg and 70 g/kg, while concentration of the feed solution was fixed at 0.5 g/kg. As shown in Figure 5a, the power density increases with both feed and draw solutions temperatures. The power density reaches 16 W/m^2^ at feed and draw temperatures of 50 °C, and a concentration of draw solution of 70 g/kg. The effect of the concentration difference between the draw and feed solutions on power density and specific entropy generation is more substantial compared to the effect of temperature. However, feed and draw solution temperatures have a significant effect on the power density and entropy generation at a high concentration difference. Moreover, the feed solution temperature has more impact on the power density and entropy generation compared to draw solution temperature. At 70 g/kg, increasing the draw solution temperature by 10 °C increased the power density by 1 W/m^2^, while increasing the feed temperature by 10 °C increases the power density by 3 W/m^2^. This is due to the fact that water permeability is strongly affected by the feed temperature. Furthermore, increasing the temperature affects the membrane structure parameter as given by Equation (16), which increases water and salt flux. The specific entropy generation increases with temperature as shown in Figure 5b. This is a result of the increase in temperature which increases the osmotic pressure of the solutions and subsequently increase the water flux.

### 4.2. Effect of Applied Pressure and Solutions Temperatures on PRO Performance

**Figure 6:**
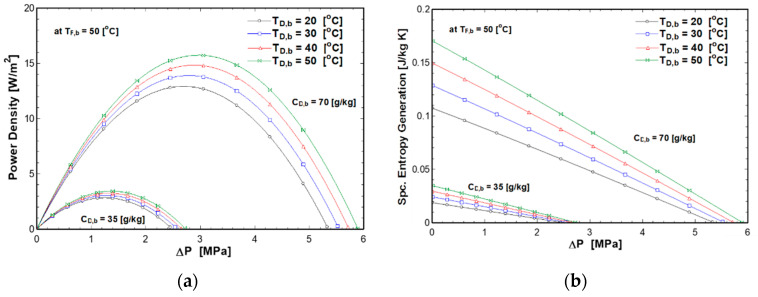
Effect of pressure and draw solution temperature on (**a**) power density (**b**) entropy generation at C_F,b_ of 0.5 g/kg and T_F,b_ of 50 °C.

**Figure 7:**
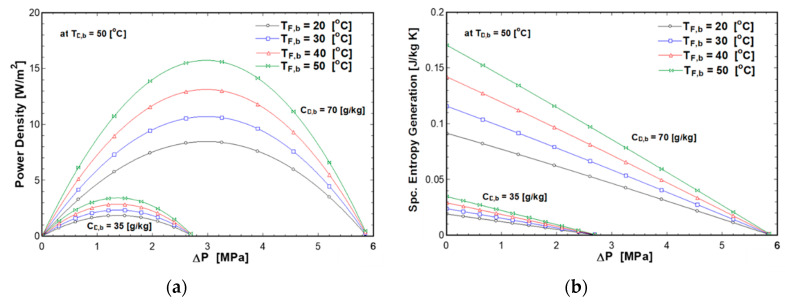
Effect of pressure and feed solution temperature on (**a**) power density (**b**) entropy generation at C_F,b_ of 0.5 g/kg and T_D,b_ of 50 °C.

This section discusses the effect of applied pressure on the power density and specific entropy generation at different feed and draw temperatures, as presented in Figure 6 and Figure 7. The pressure varied from 0 to 6 MPa. The power density increased with pressure until it reached a maximum, then it started to decrease to zero (at reversal point when ΔP=Δπ) as shown in Figure 6a and Figure 7a. Increasing the draw solution temperature increased the osmotic pressure as given in Equation (6), therefore increasing the maximum pressure at the reversal point. On the other hand, the specific entropy generation decreased until it reached zero when the pressure difference was equal to the osmotic pressure difference, as shown in Figure 6b and Figure 7b, because at this point the flux is equal to zero as determined from Equation (3). Moreover, at higher concentration, feed and draw temperatures have more effect on the power density and specific entropy generation. This is due to the high osmotic pressure difference, which increases the water flux.

### 4.3. Effect of Draw Concentration and Solutions Temperatures on PRO Performance

**Figure 8:**
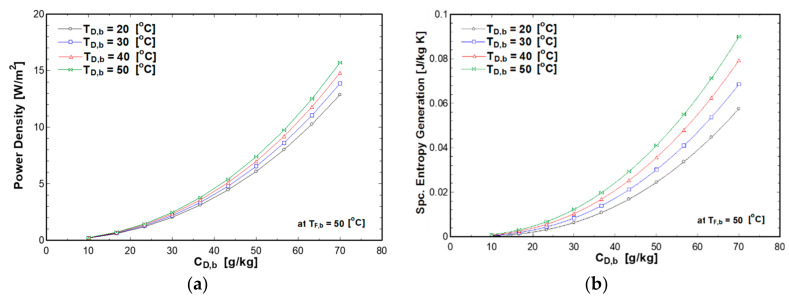
Effect of draw solution concentration and draw solution temperature on (**a**) power density (**b**) entropy generation at C_F,b_ of 0.5 g/kg and T_F,b_ of 50 °C.

**Figure 9:**
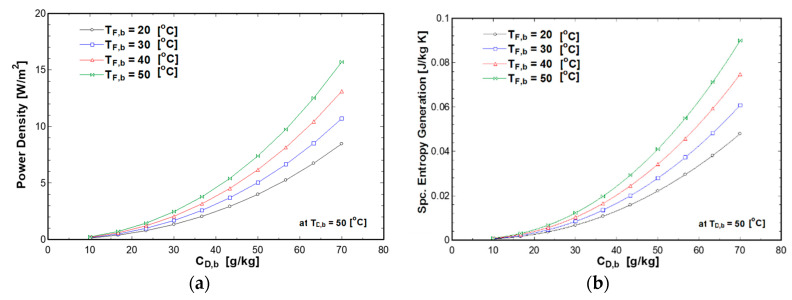
Effect of draw solution concentration and feed solution temperature on (**a**) power density (**b**) entropy generation at C_F,b_ of 0.5 g/kg and T_D,b_ of 50 °C.

Figure 8 and Figure 9 represent the effect of draw solution concentration on the power density and entropy generation with different feed and draw temperatures. The draw and feed solutions temperatures varied from 20 to 50 °C, whereas the concentration of draw solution varied from 10 g/kg to 70 g/kg. This range covers brackish water (10 g/kg), seawater (35 g/kg), and concentrated brine rejected from desalination plants (70 g/kg). The pressure difference was equal to half of the osmotic pressure difference. The results showed that the power density and specific entropy generation increased with the draw concentration. This is mainly due to the increase in osmotic pressure difference. Additionally, feed solution temperature has a significant impact on the power density and specific entropy generation at high draw concentrations. This is due to the increases of the water flux with osmotic pressure difference and feed solution temperature as given by Equations (3) and (5). On the other hand, power density and specific entropy generation slightly increased with feed temperature at a low draw concentration due to the low osmotic pressure difference.

### 4.4. Effect of Flow Rate on PRO Performance

**Figure 10:**
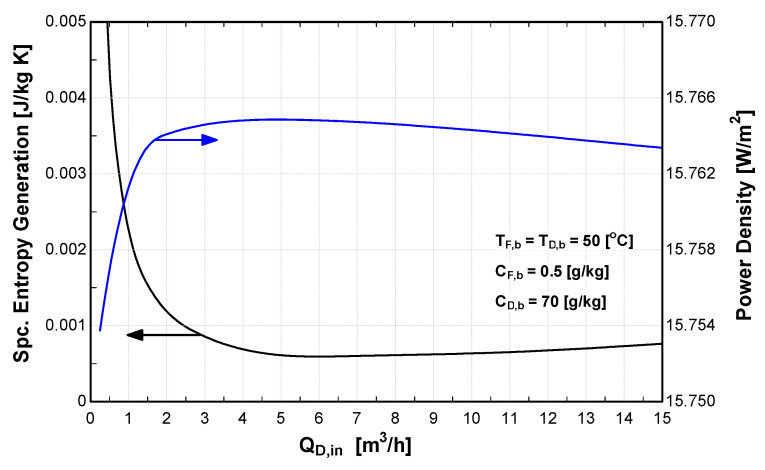
Effect of water flow rate on power density and entropy generation.

Water flow rate is one of the important parameters that affects the performance of PRO system. Thus, the performance of the PRO system is analyzed taking into consideration the effect of water flow rate as shown in Figure 10. Feed and draw solutions were maintained at a constant temperature of 50 °C, whereas the pressure difference was equal to half of the osmotic pressure difference to simulate the maximum power density at all flow rates. The feed and draw concentrations were 0.5 g/kg and 70 g/kg, respectively. It is also assumed that the feed and draw flow channels have a length of 25 mm and height of 2.5 mm as given in Table 1. The results show that the power density increases until it reaches an optimal value then starts to decrease. On the other hand, the specific entropy generation decreases until it reaches a minimum and then starts to increase. As the volumetric flow rate increases, the velocity and turbulence in the feed and draw channels increases, which increases Reynolds number and the diffusion coefficient. On the other hand, the pressure drop due to the friction and turbulence increases which increase the entropy generation and irreversibility in the process. At a high flow rate, the effect of the pressure drop becomes dominant, thus power density decreases, and specific entropy generation increases. The optimum flow rate for the investigated operating condition and flow channels dimensions was found to be 5.5 m^3^/hr as shown in Figure 10. This is corresponding to an average velocity in the flow channel of about 17.8 m/s and a Reynold number of 23,500. This is a very high velocity for a real situation and is hard to practically achieve because it is a highly turbulent flow in the channels. However, these values change with the change of the operating conditions and flow channel dimensions. The key point from Figure 10 is to show that the maximum power density is approached at the minimum entropy generation, hence minimum irreversibility in the process. Moreover, the change in the power density and entropy generation near the optimum flow is very small (the curves are flat near the optimum point). This means that the optimum power density is not very sensitive to the variation of the flow rate. 

## 5. Conclusions

A numerical model was developed to study the effect of feed solution and draw solution temperatures on the performance of PRO process. The effect of concentration polarization was considered as well as the effect of temperature on the membrane structure parameter and water properties. The results of the numerical model were in agreement with the experimental data found in the literature. The model also investigated the effects of pressure difference, temperature, concentration difference and flow rate on the power density and entropy generation. The results show that the power density strongly depends on the solution temperature especially at high concentration difference. Moreover, the feed solution temperature has a higher impact on the power density and entropy generation than that of the draw solution temperature. This is due to the effect of the temperature on the membrane structure parameter, which increases water and salt flux. The volumetric flow rate hence the flow velocity in the flow channels has an impact on the power density and entropy generation. It was found that there is an optimum flow rate value that maximizes the power density and minimizes the entropy generation when fixing other operating parameters and the flow channels dimensions. 

## Figures and Tables

**Table 1:** IGB membrane properties.

Parameter	Value
Pore size	0.35 nm
Porosity of the support layer	80%
Porosity of the active layer	0.6%
Support layer thickness	12 μm
Active layer thickness	100 nm
Effective membrane area	18 cm^2^
Channel dimensions **(for feed and draw solutions)**	40 mm–25 mm–2.5 mm
Water permeability coefficient A	1.06 × 10^−9^ m/s kPa
Salt permeability coefficient B	2.62 × 10^−8^ m/s

**Table 2:** Experimental conditions.

Variable	Value
Feed temperature	20–50 °C
Draw temperature	20–50 °C
Feed concentration	0.5 g/kg
Draw concentration	35 g/kg–70 g/kg
Volume flow rate	0.1–15 m^3^/hr

## Nomenclature

A	membrane water permeability	m/s-kPa
*A_m_*	membrane surface area	m^2^
*B*	membrane salt permeability	m/s
*C*	solute concentration or salinity	g/kg
*D*	diffusion coefficient	m^2^/s
*d_h_*	hydraulic diameter	m
*J*	flow flux	m/s
K	solute resistivity	s/m
k	mass transfer coefficient	m/s
*L*	membrane length	m
$\dot{m}$	mass flow rate	kg/s
ΔP	transmembrane pressure difference between feed and draw	kPa
$\Delta P_{drop}$	pressure drop along flow channel	kPa
*Q*	volumetric flow rate	m^3^/s
*R*	universal gas constant	J/mole K
Re	Reynolds number	-
*r_p_*	pore size	m
*S*	structure parameter	m
$S_{gen}$	specific entropy generation	J/kg K
$\dot{S_{gen}}$	rate of entropy generation	W/K
*s*	specific entropy	J/kg K
*Sc*	Schmidt number	-
*Sh*	Sherwood number	-
*T*	temperature	°C
*T_w_*	average water temperature	°C
*t_m_*	membrane thickness	m
*V*	velocity	m/s
*W*	power density	W/$m^{2}$
**Greek symbols**		
*β*	van’t Hoff coefficient	-
*ε*	porosity of the membrane	-
*μ*	viscosity	kg/ms
*π*	osmotic pressure	kPa
*ρ*	density	kg/m^3^
**Subscripts**		
*b*	bulk	
*D*	draw solution	
*F*	feed solution	
*i*	cell number	
*icp*	surface between active layer and support layer	
*m*	membrane surface	
*s*	salt or solute	
*w*	water	
